# Is a Meta-Analysis of Clinical Trial Outcomes for Ketogenic Diets Justifiable? A Critical Assessment Based on Systematic Research

**DOI:** 10.3390/foods13203219

**Published:** 2024-10-10

**Authors:** Nicole Hunter, László Czina, Edit Murányi, Balázs Németh, Tímea Varjas, Katalin Szendi

**Affiliations:** 1 Department of Public Health Medicine, Medical School, University of Pécs, 7624 Pécs, Hungary; 2 MTA–PTE Lendület “Momentum” Evidence in Medicine Research Group, Department of Public Health Medicine, Medical School, University of Pécs, 7624 Pécs, Hungary

**Keywords:** ketogenic diet, nutritional ketosis, clinical trial, meta-epidemiological assessment, systematic search, review

## Abstract

While the macronutrient content of a ketogenic diet specifically utilized for childhood epilepsy is clearly defined in the literature, variations among other ketogenic diets exhibit substantial heterogeneity. Furthermore, studies utilizing ketogenic diets contain several confounders with notable impacts on outcomes, thereby rendering both their findings and those of the meta-analyses less reliable. The objective of this meta-epidemiological assessment was to scrutinize existing clinical trials that investigated the effects of ketogenic diets on patients with obesity and diabetes, thereby determining the feasibility of conducting a meta-analysis. The Ovid Medline, Scopus, Cochrane Central Register of Controlled Trials (CENTRAL), and Embase databases were searched from 1946 to 24 September 2024. Of the studies reviewed, none met the predefined inclusion criteria. However, seven articles met these criteria very closely. In the future, studies investigating the effects of ketogenic diets containing significant confounding factors should adopt a single definition of a ketogenic diet. Additionally, accurate measurement of actual macronutrient and caloric intake, along with regularly monitored nutritional ketosis, will be essential to highlight the true effects of a ketogenic diet.

## 1. Introduction

The classic therapeutic ketogenic diet (KD) (fat = 90%, protein = 6%, carbohydrate = 4%) with no caloric restriction [1] was established in 1921 as an effective nonpharmacological treatment for refractory childhood epilepsy [2]. The presence of ketone bodies was checked daily in children treated with a KD so that their statuses could be accurately monitored [3].

Since 1921, various types of KDs have become increasingly popular among those aiming to lose weight. Although KDs are currently considered a “fad diet” rather than a scientifically proven healthy eating regimen, the results of meta-analyses generally suggest positive health effects of KDs [4]. In our previous work, we conducted a critical literature review of meta-analyses [4] summarizing the effects of KDs. The review revealed that these analyses and, thus, the clinical trials they encompass were affected by numerous confounders, significantly influencing the results of meta-analyses on the effects of KDs. This is not only problematic given that many meta-analyses yield favorable outcomes regarding KDs, which can be misleading to both lay audiences and professionals, but, also, questions the integrity of these studies given that meta-analyses typically provide reliable, high-level evidence [4]. However, if clinical trials contain significant confounding factors, the results of the meta-analyses cannot be considered reliable.

KDs can take many forms, but they must share one key common factor: the presence of ketone bodies (ß-hydroxybutyrate [BHB]) in the blood within the range of 0.5–3.0 mmol/L, which is called the range of nutritional ketosis [5,6]. If patients are not in a state of nutritional ketosis, then the diet cannot be considered a KD.

Furthermore, merely specifying a daily carbohydrate intake of <50 g in a clinical trial is inadequate, as the threshold for inducing nutritional ketosis differs among individuals [7]. Therefore, continuous monitoring for the presence of nutritional ketosis on an individual basis is necessary.

However, even this factor (nutritional ketosis) does not precisely define what a KD is. This begs the following question: What do we mean by a KD? Is there any way to induce nutritional ketosis other than through the Harvard University definition of a KD [8]? The short answer is that yes, it is possible. However, the caveat is that the patient will be starving as a result of following a very-low-calorie diet (600–800 kcal/day). Would we call a diet with 600–800 kcal/day ketogenic simply because patients are starving? Does starvation equal a KD? Therefore, is everyone who is starving on a KD?

According to the Harvard University definition of a KD, 70–80% of the total daily caloric intake should come from fat, 5–10% from carbohydrates, and 10–20% from protein. For a 2000 kcal diet, this means approximately 165 g of fat, 40 g of carbohydrates, and 75 g of protein [8]. The National Cancer Institute’s definition is much less precise and informative (“a diet high in fat and low in carbohydrates” and mentions ketosis) [9].

Approximately 90% of patients with type 2 diabetes are overweight or obese [10]. Obesity is a major risk factor in developed countries [11]. Numerous individuals seek to shed excess weight through various means whether for esthetic or health reasons. Among the most popular weight loss strategies today, the KD stands out as one of the most widely embraced. Consequently, a considerable number of individuals are impacted by the outcomes of scientific inquiries into its efficacy.

The objective of this meta-epidemiological assessment was to scrutinize existing clinical trials that investigated the effects of KDs on patients with obesity and diabetes. Our aim was to identify any confounding factors outlined in our prior research [4] and ascertain the feasibility of conducting a meta-analysis meeting the following criteria: (i) adherence to a normocaloric diet and equivalence in caloric intake between the KD group and the control group; (ii) precise definition of the KD in terms of macronutrient proportions (g/%), which mostly match the values defined by Harvard University [8]; (iii) regular monitoring of diet adherence throughout the study with periodic assessment of ketone body levels to confirm nutritional ketosis; (iv) emphasis on the consumption of saturated or unsaturated fatty acids; and (v) inclusion of kidney and liver function measurements. In addition, longer-term studies would be more informative [4].

## 2. Materials and Methods

### 2.1. Protocol

A meta-epidemiological assessment based on systematic research was used to compile all relevant evidence provided by interventional studies on the effects of KDs on patients (aged > 18 years) with obesity and diabetes conducted between 1946 and 2024.

### 2.2. Eligibility Criteria

To be included in this meta-epidemiological assessment, a study had to fulfill the following criteria based on our previous review [4]: (i) it had to be a prospective interventional clinical trial; (ii) it needed to focus on the effects of a low-carbohydrate, high-fat diet; (iii) the macronutrient ratios actually consumed had to align with those defined by Harvard University (fat: 70–80% (165 g); carbohydrate: 5–10% (20–50 g); protein: 10–20% (75 g), ~2000 kcal), or fat could be 60–75% of calories if a high proportion of medium-chain triglycerides are consumed. In this case, the proportion of protein and carbohydrates could be increased [12]; (iv) caloric intake equivalence was required between the KD group and the control group; (v) regular assessment of ketone body levels in the blood or urine was necessary to confirm nutritional ketosis for the duration of the study; (vi) the study population had to consist of adult patients (aged > 18 years) with metabolic syndrome, overweight, obesity, or type II diabetes; (vii) outcomes measured included weight loss and lipid and glucose parameters in the blood; and (viii) the results had to be published between 1946 and 2024. There were no language restrictions in the database search.

The authors chose to exclude articles on a KD when (i) the KD was examined in a patient population performing resistance training; (ii) the patients were athletes; or (iii) the patients had cancers, neurological disorders, or other diseases. Therefore, even if weight loss was an outcome of a study, we excluded it, as our focus was solely on examining the impact of the KD in an overweight/obese population seeking weight reduction. Very-low-calorie ketogenic diets (VLCKDs) (<800 kcal) and other types of KDs were also excluded because their macronutrient ratios differed significantly from the KD definition of Harvard University [8].

### 2.3. Search Strategy

The Ovid Medline, Scopus, Cochrane Central Register of Controlled Trials (CENTRAL) and Embase databases were searched from 1946 to 7 March 2024. Text words with appropriate truncation and relevant indexing terms for “ketogenic diet/low carbohydrate diet” were used. The language of publication was not a limiting factor during research collection. The electronic searches were updated on 24 September 2024. The final search strategy for Ovid MEDLINE can be found in Supplementary Material S1.

Additional articles were obtained through manual searches of the meta-analyses selected in our previous study [4].

### 2.4. Screening and Data Extraction

Each reference was screened by two independent reviewers based on predefined inclusion criteria via Covidence online software (Veritas Health Innovation, Covidence systematic review software. Melbourne, Australia). First, the titles and abstracts of the studies were screened to exclude irrelevant references. Second, the full texts of potentially relevant studies were checked for final inclusion. Five authors (KS, EM, TV, BN, and NH) were involved in the title and abstract screening process.

One author (KS) developed the data-charting form to determine which variables to extract. Three authors (KS, EM, NH) independently charted the data, discussed the results, and continuously updated the data-charting form. Items were selected for charting in MS 365 Excel 2409 (build: 18025.20104). The following data were extracted from each included study by one reviewer and checked for accuracy by a second reviewer: bibliographic details (first authors and year), disease/test parameters, duration of the experiment, characteristics of the experimental and control groups (macronutrient ratio and caloric intake), adherence to the diet, consumed type of fatty acid, measurement of ketone bodies, liver and kidney function measurement, and positive or negative results.

## 3. Results

### 3.1. Selection of Sources of Evidence

**Figure 1:**
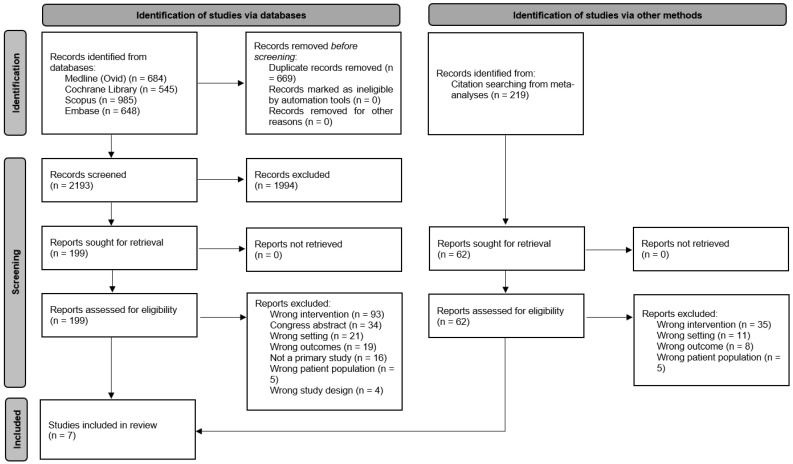
PRISMA flow diagram for new systematic reviews including searches of databases, registers, and other sources [13].

Figure 1 shows the selection process using a PRISMA flow diagram [13].

### 3.2. Characteristics of Sources of Evidence

The study characteristics are presented in Table 1, which contains data on the name of the first author; disease/test parameters; duration of the experiment; planned (and real) macronutrient ratio (%) of the experimental and control groups; controlling and monitoring of food intake; average real caloric intake of the experimental and control groups; consumed type of fatty acid; results of ketone body measurements; liver and kidney function measurements; and outcomes.

### 3.3. Results of Individual Sources of Evidence

In most of the articles selected for full-text analysis (199 from Covidence and 62 from meta-analyses), there was no consistent definition of a KD. It was found that, with few exceptions, studies failing to follow the macronutrient guidelines set by Harvard University led to participants not reaching nutritional ketosis. In other words, the diet was not truly ketogenic. The authors of this meta-epidemiological assessment excluded VLCKD studies for the reasons described in the Introduction and Eligibility Criteria section (starvation is a confounding factor when studying the effects of KDs).

While none of the studies met the predefined inclusion criteria, seven articles were very close to the outlined criteria [14,15,16,17,18,19,20] (Table 1).

We included seven studies with 161 participants. All the seven studies were randomized controlled interventional clinical trials (RCTs). Five studies were conducted in the USA [14,15,16,18,20], one in the United Kingdom [17] and one in China [19]. Although the actual macronutrient intake rates according to the Harvard definition differed slightly (with no available data for Buga et al. [14] and White et al. [20]), food intake was strictly controlled and closely monitored in these seven studies. In six articles [14,15,16,17,18,20], the food was prepared for the participants; in one article [19], the participants were instructed to measure urinary ketone levels daily; and in two articles [14,18], the participants reported BHB daily. For ketone body levels, values in mmol/L were provided in six articles [14,15,16,18,20]. In one article [19], the percentage of days the participants were in ketosis was given, but the specific values were not published. The average caloric intake in four studies [15,17,18,19] was lower in the KD group than in the control group, which may have led to inaccurate results. In White et al.’s article [20], the only information on calorie intake was that it was “70% of that needed for weight maintenance”, and in Buga et al.’s article [14] it was “75% of estimated energy requirements for weight-maintenance”, with no measured data provided. In Hall et al.’s article [16], the average caloric intake was lower in the control group than in the KD group. None of the seven articles emphasized the importance of unsaturated fat intake over saturated fat intake in KDs. In one article [20], there was no information on the type of fat consumed. These seven studies examined the short-term effects (2–6 weeks) of a KD, and no major differences in kidney or liver function were expected within this period. Three studies [16,17,20] conducted renal function measurements, and one study [15] measured liver function. With respect to outcomes, Buga et al. [14], Crabtree et al. [15], Hall et al. [16], and Kackley et al. [18] reported similar/significant weight loss in both groups, Johnstone et al. [17] reported favorable effects of a KD, Sun et al. [19] reported both favorable and neutral results, and White et al. [20] reported neutral and unfavorable results. Sun et al. [19] were the only ones to highlight one of the key aspects of the KD: “with unchanged calorie intake, the weight-loss effects should be interpreted as resulting from changes in macronutrient composition”.

Most of the full-text papers did not measure ketone bodies at all [6,21,22,23,24,25,26], or if they did, they failed to report the results [27,28,29,30,31,32,33]. In cases where results were reported, not all patients on a KD were in nutritional ketosis for the entire duration of the study [7,34,35,36,37,38,39].

Most of the screened full-text papers did not use the Harvard University definition of KD. However, some articles did adhere to these macronutrient ratios [22,24,32,38,40,41,42]. For these articles, it would have been crucial to know whether the patients were actually able to maintain the predetermined macronutrient ratios throughout the nutritional intervention and whether the patients were in nutritional ketosis.

Among the full texts reviewed, none explicitly emphasized the importance of replacing fat with unsaturated, nonanimal fatty acids, despite the well-known adverse health effects of saturated fats. Additionally, we did not find any studies on plant-based diets adhering to a ketogenic regimen considering our inclusion criteria.

## 4. Discussion

To the best of the authors’ knowledge, this is the first meta-epidemiological assessment based on systematic research that scrutinizes the significant confounding factors influencing studies on the effects of KDs and which questions the validity of meta-analyses and the reliability of their results.

The main confounding factors identified in the current assessment and observed in a significant portion of the reviewed articles were (i) macronutrient ratios that were altered from the planned values; (ii) the absence of nutritional ketosis; and (iii) starvation (predominantly in VLCKD studies).

Owing to the actual, altered macronutrient ratios reported in these studies, patients either failed to reach the range of nutritional ketosis or it was not measured at all by the researchers. Thus, altered macronutrient ratios which are not followed and no information on nutritional ketosis are confounding factors. Starvation resulting from very-low-caloric intake in VLCKD studies should not necessarily be classified as a KD. Nonetheless, it remains a popular choice among the various types of KDs examined. If someone follows a VLCKD (600–800 kcal/day), one cannot be certain whether the resulting nutritional ketosis occurred because of the KD itself or because of starvation. In this case, low-caloric-intake-induced starvation is a confounding factor. Moreover, in the case of VLCKDs, the macronutrient ratios defined by Harvard University cannot be maintained. KDs represent a relatively recent trend in dieting, yet starvation is not a novel approach to weight management. Limiting calorie intake is not exclusive to any particular diet. Insufficient caloric intake (25% reduction in energy intake below baseline levels) from any source can induce starvation, leading to ketone body production [43], weight loss, and improvement in blood sugar and lipid parameters. This could have happened in the trials of Buga et al. [14] and White et al. [20]. Therefore, when examining KDs, a distinction should be made between nutritional ketosis due to starvation and nutritional ketosis due to a normocaloric (~2000 kcal) KD as defined by Harvard University [8].

To monitor weight loss, blood glucose, and blood lipid parameters effectively while excluding confounding factors and achieving nutritional ketosis, it is essential to adhere to a standardized definition of the KD. The definition provided by Harvard University could serve this purpose well, as the authors have not found a more precise and reliable definition to date. This standardization would be crucial because most other forms of the KD (e.g., the Atkins diet or modified Atkins diet) do not consistently induce nutritional ketosis, rendering the results of such studies irrelevant to the true KD. Additionally, if both groups are normocaloric and follow the same caloric intake, the impact of extreme macronutrient ratios specific to the KD can be assessed without the influence of confounding factors. This perspective is also supported by Sun et al. [19].

Based on our experience from current and previous [4] literature reviews, the following elements would be beneficial for an interventional study.

KD definition: Instead of relying on the many different and often incomparable KD definitions that include numerous confounding factors, it would be beneficial to use a single standard, such as the Harvard definition [8], which outlines specific macronutrient ratios and recommended caloric intake.

Adherence: It would be advantageous to measure the predefined dietary factors (macronutrient ratios and calorie intake) during the study. This would provide information on the actual amounts consumed. Studies suggest that adherence is highest when all food to be consumed is prepared in advance and closely monitored. Without this control, participants often fail to maintain the prescribed caloric intake and macronutrient ratios. As a result, they are neither on a KD nor in nutritional ketosis.

Nutritional ketosis: Maintaining nutritional ketosis throughout the study is crucial. The effects of a KD can be evaluated only if patients remain within the range of nutritional ketosis for the entire duration of the study. Various methods have been described in the literature to measure this range, including blood, urine, and even home tests. Ideally, measurements should be taken daily, most likely after food intake.

Calorie intake: The experimental and control groups should have the same, nonreduced caloric intake. This ensures that any improvements in weight loss and other related parameters are not due to caloric reduction (a confounding factor) but are solely because of the KD.

Types of fatty acids: An appropriate proportion of unsaturated fatty acids should be recommended as part of a KD. Since fat constitutes the majority of macronutrient intake, consuming healthy fatty acids could be significant in reducing cardiovascular risk factors in a long-term study.

Liver and kidney function: Monitoring renal and hepatic function is also essential in all cases of extreme macronutrient shifts, especially in long-term studies.

## 5. Limitations

This meta-epidemiological assessment evaluates the key confounding factors that can significantly impact the accuracy and reliability of clinical research findings on KDs. While this review focuses on major confounders, it acknowledges that other factors may exist which were not discussed.

Had we included studies examining the effects of KDs not only on individuals with obesity or overweight but also on those with other conditions (e.g., cancer) or different populations (e.g., athletes), we might have identified studies that met the inclusion criteria. Similarly, if we had explored the relationship between KDs and epilepsy, we would almost certainly have found relevant studies, as the effectiveness of KDs in managing epilepsy symptoms is closely tied to continuous monitoring of nutritional ketosis.

However, this meta-epidemiological analysis is specifically concerned with assessing the reliability of sources regarding the use of KDs for weight loss, since overweight and obesity are widespread challenges in developed nations, and ketogenic diets have gained popularity as a weight loss strategy.

## 6. Conclusions

The authors believe that KDs, with their macronutrient ratios deviating significantly from the WHO recommendations for healthy eating [44], and their high intake of saturated fats, cannot be considered a healthy dietary alternative. However, the authors remain open-minded and are interested in reviewing interventional studies that are free from the discussed significant confounders.

This meta-epidemiological assessment critically evaluates the existing body of research surrounding KDs, particularly focusing on their application in obesity and diabetes management. Our findings highlight the significant confounding factors in RCTs that compromise the reliability of current meta-analyses, primarily stemming from inconsistent adherence to defined macronutrient ratios, inadequate monitoring of nutritional ketosis, and the influence of starvation conditions in VLCKDs. Consequently, conducting a meta-analysis of these studies at this time is not advisable, as it could result in misleading conclusions.

To ensure the validity and integrity of future studies, it is imperative that researchers adhere to a standardized definition of KDs, such as the Harvard University guidelines, which stipulate specific macronutrient ratios and caloric intake. Furthermore, continuous monitoring of diet adherence and ketone body levels is crucial to differentiate the effects of a genuine ketogenic diet from those that have not been validated as ketogenic through regular ketone body assessments.

Our review underscores the necessity for more rigorous long-term studies that prioritize proper dietary controls and measurements of kidney and liver function. Such approaches will facilitate a clearer understanding of the health implications of KDs and their effectiveness as therapeutic interventions for individuals with obesity and metabolic disorders. Ultimately, the establishment of these methodological standards is crucial not only for advancing the scientific rigor of ketogenic diet research but also for informing clinical practice and public health recommendations.

## Figures and Tables

**Table 1:** Extracted data from articles that closely met the predefined inclusion criteria for this assessment.

Reference, Type of Clinical Trial	Disease/Test Parameters	Duration (Day/Week)	Planned (and Real) Macronutrient Ratio (%/g) of Experimental Group	Planned (and Real) Macronutrient Ratio (%) of Control Group	Controlling and Monitoring of Food Intake	Average of Real Calorie Intake of Experimental Group	Average of Real Calorie Intake of Control Group	Fatty Acid Type	Results of Ketone Body Measurements	Liver and Kidney Function Measurement	Outcome
Buga et al. [14], RCT	Overweight and obesity	6 weeks	Ketogenic diet (KD): Carbohydrate <50 g, protein 1.5 g/kg of ideal bodyweight; the remainder of individual energy needs were derived from lipids (No data)	Low-fat diet (LFD): USDA’s Dietary Guidelines for Healthy Americans 2015–2020 (No data)	All the food was prepared that was provided to participants. Ingredients were precisely weighed on electronically calibrated scales.	75% of estimated energy requirements for weight-maintenance	75% of estimated energy requirements for weight-maintenance	Emphasis on monounsaturated and saturated fat sources. KD group: + two servings of 10 g medium-chain fatty acid (MCT) oil	Nutritional ketosis was attained after day 5 (1.0 ± 0.04 mM BHB) in the KD group (daily fasting capillary ketone measurement). LFD had no impact on BHB.	No data.	Similar weight loss in both groups.
Crabtree et al. [15], RCT	Overweight	6 weeks	Ketogenic diet (KD): No data (Carbohydrate 38 ± 7 g, fat 131 ± 8 g, protein 100 ± 3 g)	Low-fat diet (LFD): No data (Carbohydrate 259 ± 8 g, fat 51 ± 9 g, protein 100 ± 3 g)	Each meal was prepared and provided to the participants. Each ingredient was precisely weighed (±0.1 g) with custom macro-and micronutrient composition personalized to each participant.	1752 ± 98 kcal (75% of energy expenditure)	1900 ± 102 kcal (75% of energy expenditure)	Rich in saturated fat	The KD group reached the 0.5 mM threshold indicating ketosis by day 3 and remained in nutritional ketosis throughout the intervention (1.12 ± 0.12 mmol/L BHB).	There were no differences in other major liver function enzymes (AST, ALT, AST/ALT, and bilirubin) from baseline to post-intervention.	Weight loss was similar between groups. Hypocaloric low-fat diet and KD can both be used in the short term to significantly reduce liver fat in individuals with NAFLD.
Hall et al. [16], RCT	Overweight	2 weeks	Animal-based, ketogenic, low-carbohydrate diet (LC): No data (Carbohydrate 10.0%, fat 75.8%, protein 14.2%)	Plant-based, low-fat diet (LF): No data (Carbohydrate 75.2%, fat 10.3%, protein 14.5%)	Inpatient study. Participants were presented with three daily meals and a continuous supply of snacks and bottled water.	Ad libitum	Ad libitum (689 ± 73 kcal d^−1^ less energy intake)	Animal-based	Daily capillary BHB: average concentration of 1.8 ± 0.1 mM. Daily urinary excretion of ketones was 1.44 ± 0.06 g d^−1^.	Daily excretion of urea was lower during the LF diet, as was excretion of ammonia and creatinine.	LF diet led to 689 ± 73 kcal d^−1^ less energy intake than the LC diet. Body weight decreased during both diets. Total cholesterol, LDL, and HDL were significantly higher in the LC group compared to the LF group.
Johnstone et al. [17], RCT	Obesity	4 weeks	Low-carbohydrate ketogenic diet (LC): Carbohydrate 4%, fat 66%, protein 30% (Carbohydrate 5%, fat 66%, protein 30%)	Medium-carbohydrate nonketogenic diet (MC): Carbohydrate 35%, fat 35%, protein 30% (Carbohydrate 36%, fat 34%, protein 30%)	Food was provided daily. Food was weighed before and after consumption to measure intake. Subjects completed food diaries.	1732 kcal	1900 kcal	Mostly saturated fats	Mean 3-hydroxybutyrate concentrations: 1.52 mmol/L in plasma and 2.99 mmol/L in urine. All subjects became ketotic after 1–3 d of the LC diet and remained so for the duration of the dietary period.	No significant alteration in plasma urea.	Energy intake and hunger was significantly lower and weight loss was significantly greater with the LC diet.
Kackley et al. [18], RCT	Overweight/Obesity	6 weeks	Ketogenic diet (KD): Carbohydrate 40 g/day (Carbohydrate 9%, fat 67%, protein 23%)	Low-fat diet (LFD): Fat 25% (Carbohydrate 55%, fat 24%, protein 21%)	All the food was prepared in a metabolic kitchen and 100% of the food containers were empty for both the LFD and KD when returned to the testing facility. Daily blood ketones were measured.	1752 ± 350	1900 ± 296	Emphasis on monounsaturated and saturated fat sources	KD elevated R-βHB into the range of nutritional ketosis (>0.5 mM *R*-βHB) throughout the experiment.	No data.	Both diets elicited clinically significant weight loss and improved insulin sensitivity and serum lipids. Fasting plasma glucose and inflammatory markers were not different between diets.
Sun et al. [19], RCT	Overweight	4 weeks	Low-carbohydrate diet (LC): Carbohydrate 10%, fat 65%, protein 25% (Carbohydrate 9.3 ± 5.5%, fat 68.1 ± 4.6%, protein 22.8 ± 3.2%)	Control diet (CON): “Normal diet” (Carbohydrate 43.1 ± 7.9%, fat 40.2 ± 5.7%, protein 15.9 ± 3.6%)	Food records for 2 weekdays and 1 weekend day. Self-testing reagent strips to measure urinary ketones daily in the early morning or after dinner. Weekly follow-up.	1776 ± 284 kcal	1990 ± 345 kcal	Types of fat from saturated or unsaturated sources were not restricted	Urinary ketosis was detected on 97.6 ± 4.5% of the days in the LC group.	No data.	Significant reductions in body weight, BMI, and waist-to-hip ratio in the LC group. Fasting glucose and blood lipid levels remained unchanged in groups.
White et al. [20], RCT	Overweight	14 days	Ketogenic diet: Carbohydrate 5%, fat 65%, protein 30% (No data)	Nonketogenic diet: Carbohydrate 40%, fat 30%, protein 30% (No data)	Participants were served a hot lunch daily Monday through Friday; all other meals and snacks were packaged and consumed at home. Energy intake was strictly controlled.	70% of that needed for weight maintenance	70% of that needed for weight maintenance	No data	Beta-hydroxybutyrate concentration at week 2: 0.72 ± 0.18 mmol/L.	Elevations in urinary urea.	No difference in weight loss or reductions in fat mass between groups. Ketogenic diet enhances fatigability and can reduce the desire to exercise.

## Data Availability

No new data were created or analyzed in this study. Data sharing is not applicable to this article.

## Supplementary Materials

The following supporting information can be downloaded at https://www.mdpi.com/article/10.3390/foods13203219/s1. Supplementary Material S1: search strategy.
