# Supplementary material for: Is a Meta-Analysis of Clinical Trial Outcomes for Ketogenic Diets Justifiable? A Critical Assessment Based on Systematic Research
Source: Foods. 2024 Oct 10;13(20):3219. doi: 10.3390/foods13203219 (PMC11506855; doi:10.3390/foods13203219)
Supplement: Supplementary file 1 [file foods-13-03219-s001.zip › foods-3231723-supplementary.pdf]

Search strategy

Ovid MEDLINE® ALL <1946 to March 06, 2024>

1 (obes* or overweight* or "over weight*").ti,ab.

2 (change adj3 ((anthropometric* and measure*) or "anthropometric measure*" or (body and composition*) or "body composition*")).ti,ab.

3 (BMI* or "BMI").ti,ab.

4 ((weight and los*) or "weight los*").ti,ab.

5 Obesity/

6 Metabolic Syndrome/

7 Overweight/

8 Weight Loss/

9 Body Mass Index/

10 (blood and (lipid* or fat*)).ti,ab.

11 1 or 2 or 3 or 4 or 5 or 6 or 7 or 8 or 9 or 10

12 diabet*.ti,ab.

13 Diabetes Mellitus/

14 12 or 13

15 ((ketogen* and diet*) or "ketogen* diet*").ti,ab.

16 exp Diet, Ketogenic/

17 15 or 16

18 11 and 17

19 14 and 17

20 18 or 19

21 randomized controlled trial.pt.

22 controlled clinical trial.pt.

23 randomi?ed.ab.

24 placebo.ab.

25 clinical trials as topic.sh.

26 randomly.ab.

27 trial.ab.

28 groups.ab.

29 21 or 22 or 23 or 24 or 25 or 26 or 27 or 28

30 exp animals/ not humans.sh.

31 29 not 30

32 20 and 31

33 exp cohort studies/ or exp epidemiologic studies/ or exp clinical trial/ or exp evaluation studies as topic/ or exp statistics as topic/

34 ((control and (group* or study)) or (time and factors) or program or survey* or ci or cohort or comparative stud* or evaluation studies or follow-up* or time series).mp.

35 33 or 34

36 (animals/ not humans/) or comment/ or editorial/ or exp review/ or meta analysis/ or consensus/ or exp guideline/

37 hi.fs. or case report.mp.

38 36 or 37

39 35 not 38

40 20 and 39

41 32 or 40
